# Oscillatory shear stress-induced downregulation of TET1s injures vascular endothelial planar cell polarity by suppression of actin polymerization

**DOI:** 10.1063/5.0141289

**Published:** 2023-07-31

**Authors:** Kai Qu, Caihong Wang, Lu Huang, Xian Qin, Kun Zhang, Juhui Qiu, Guixue Wang

**Affiliations:** 1 Key Laboratory for Biorheological Science and Technology of Ministry of Education, State and Local Joint Engineering Laboratory for Vascular Implants, Bioengineering College of Chongqing University, Chongqing, China; 2 Clinical Research Center for Endocrinology and Metabolic Diseases, Chongqing University Three Gorges Hospital, Chongqing, China; 3 School of Advanced Manufacturing Engineering, Chongqing University of Posts and Telecommunications, Chongqing, China; 4 Jinfeng Laboratory, Chongqing, China

## Abstract

Vascular endothelial polarity induced by blood flow plays crucial roles in the development of atherosclerosis. Loss of endothelial polarity leads to an increase in permeability and leukocyte recruitment, which are crucial hallmarks of atherosclerotic initiation. Endothelial cells exhibit a morphological adaptation to hemodynamic shear stress and possesses planar cell polarity to the direction of blood flow. However, the mechanism of how hemodynamic shear stress regulates endothelial planar cell polarity has not been firmly established. Here, we found that TET1s, a short isoform of Tet methylcytosine dioxygenase 1, was a mediator in the regulation of the planar cell polarity in endothelial cells in response to hemodynamic shear stress. In the process, low expression of TET1s induced by oscillatory shear stress led to the endothelial planar polarity damage through inhibition of F-actin polymerization. TET1s can regulate demethylation level of the sFRP-1 promoter to alter the expression of sFRP-1, which affects the interaction of sFRP-1/Fzd4 and F-actin polymerization. Our study revealed the mechanism of how TET1s mediates endothelial planar cell polarity in response to hemodynamic shear stress and provides a new insight for the prevention of atherosclerosis.

## INTRODUCTION

Atherosclerosis is a chronic inflammatory disease that results in angina, myocardial infarction, or ischemic stroke, which are the leading causes of morbidity and mortality worldwide.^1^ Vascular endothelial cells (ECs) play a central role during atherosclerosis development from the initial to the advanced stage. Endothelial polarity is required for maintaining endothelial homeostatic functions, including shear stress-regulated mechanotransduction and anticoagulant and barrier function.^2^ Loss of endothelial polarity leads to an increase in permeability and leukocyte recruitment and contributes to the atherosclerotic development.^3^

Cell polarity refers to the uneven distribution of some cytoplasmic components in spatial order, resulting in an asymmetric organization of cellular components such as organelles and cytoskeleton.^4,5^ It is suggested that inherent cell asymmetry is essential for the function of a particular cell type.^6^ Cell polarity predominantly is of two types. The first type is the apico-basal polarity (ABP), and the other type is the planar cell polarity (PCP), in which polar cells are aligned across a common plane perpendicular to the apico-basal axis.^7^

ECs line the inner blood vessel wall and play crucial roles in the homeostasis of the circulatory system.^8^ ECs exhibit a morphological adaptation to hemodynamic shear stress and possesses PCP to the direction of blood flow.^9–11^ Hemodynamic shear stress consists of two types: laminar shear stress (LSS), which supports endothelial health and maintains vascular stability,^12^ and oscillatory shear stress (OSS), which disrupts endothelial function and contributes to the progression of atherosclerosis.^13,14^ Increasing studies showed that endothelial polarity is regulated by flow shear stress (FSS), and LSS promotes the formation of endothelial PCP.^15–18^ However, the effect of OSS on endothelial PCP is still unclear.

Tet methylcytosine dioxygenase 1 (TET1) regulates genes expression through mediating DNA demethylation by oxidizing 5-methylcytosine (5mC) to 5-hydroxymethylcytosine (5hmC), ultimately leading to DNA demethylation.^19,20^ Furthermore, recent studies reported a novel short isoform of TET1 (termed TET1s), which is a primary isoform of TET1, in most somatic tissues that exhibits a different expression and functional patterns from that of full-length TET1 (termed TET1-FL).^21,22^ Our previous studies have shown that TET1s is involved in protecting the vascular endothelial barrier, the expression of which is regulated by different shear stress. In the study, we found LSS induces TET1s expression to protect the vascular endothelial barrier by increasing CX40 expression in ECs. However, TET1s deficiency impairs the vascular intimal barrier and exacerbates oscillatory shear flow-induced atherosclerosis.^23^ Recent study found that TET1 mediates Pg. LPS/IFN-γ induced M1 macrophage polarization through the NF-κB pathway in THP-1 cells.^24^ We also found that the important roles of TET1s was to regulate endothelial polarity in response to FSS. However, the details of the mechanism is still unclear.

Here, we investigated the role of TET1s in the regulation of the endothelial PCP in response to FSS *in vivo* and *in vitro*. We demonstrate that OSS impair the endothelial PCP compared with LSS. Our results show that TET1s is a key factor in OSS-induced endothelial PCP damage by inhibiting F-actin polymerization, and sFRP-1/Fzd4 signaling pathway mediates the TET1s-induced F-actin polymerization in response to FSS.

## RESULT 1: OSS INHIBITED VASCULAR ENDOTHELIAL PLANAR CELL POLARITY *IN VIVO*

**FIG. 1.**
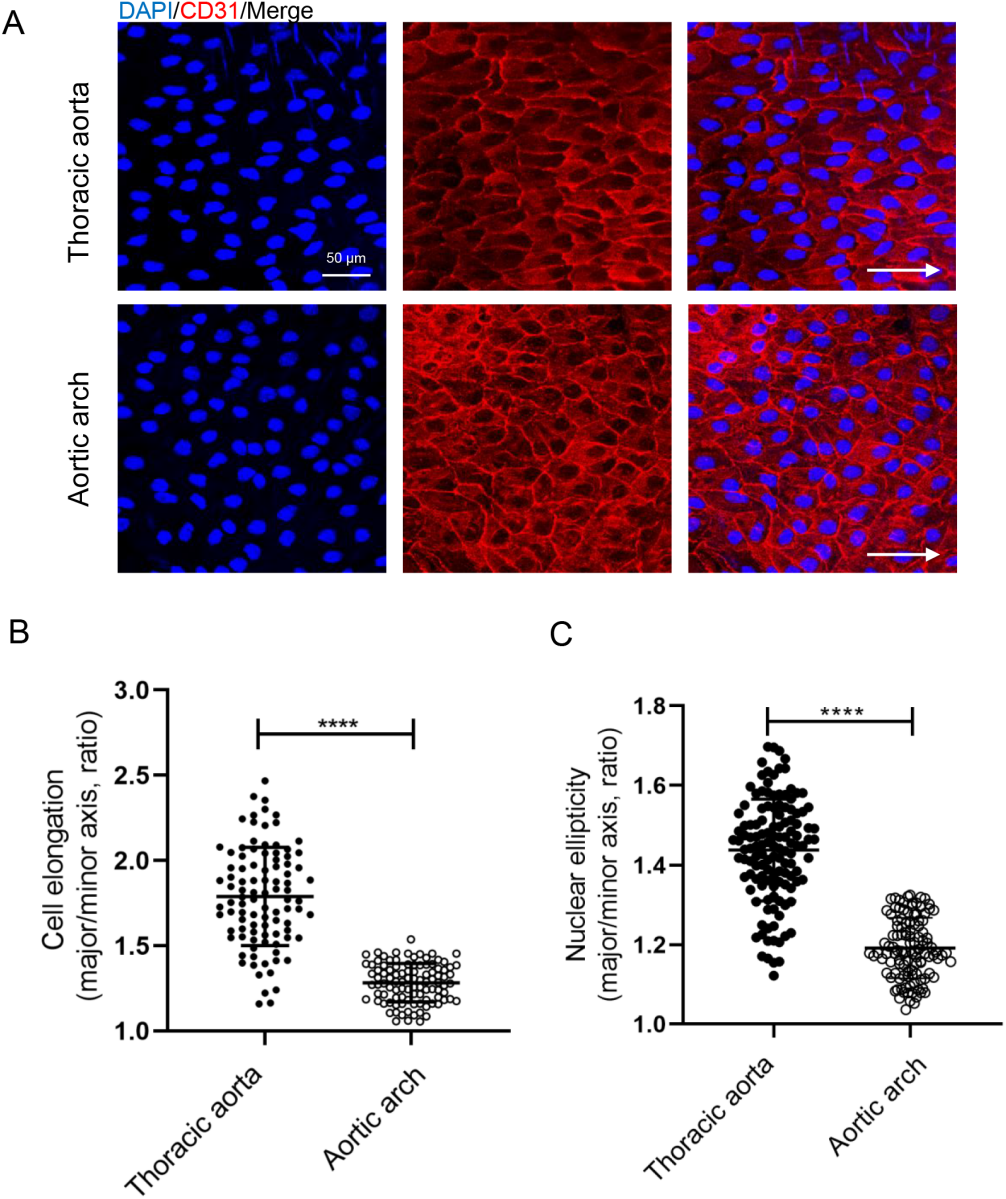
OSS inhibited endothelial cell elongation *in vivo*. (a) Immunofluorescence staining and *en face* for CD31 (red) and nuclei (blue) in ECs of thoracic aorta and aortic arch from C57BL/6J mice (WT mice). White arrows pointed in the direction of blood flow (scale bar = 50 *μ*m). (b) Statistical analysis of the elongation (the ratio of cellular major axis/minor axis) as shown in A (n > 90). (c) Statistical analysis of nuclear ellipticity (ratio of nuclear major axis/minor axis) (n > 110). All data were presented as mean ± SD. ^***^ P < 0.0001 and ^****^ P < 0.0001.

The ECs were stimulated by OSS in the inner curvature of aortic arch of mice and by LSS in the thoracic aorta.^30^ The effect of OSS on PCP of ECs was investigated by comparing endothelial morphological difference between aortic arch and thoracic aorta. We found that ECs of thoracic aorta were elongated and aligned with the axis of the vessel in the direction of blood flow [Fig. 1(a)]. Based on the direction of blood flow, the nucleus is located downstream of the long axis of the cell [Fig. 1(a)]. The morphology of aortic arch ECs was significantly different. Compared with thoracic aortic ECs, ECs in the inner curvature of the aortic arch have a decreased elongation ratio [Figs. 1(a) and 1(b)], a decreased nuclear ellipticity, and an increased cell density [Figs. 1(a) and 1(c)].

**FIG. 2.**
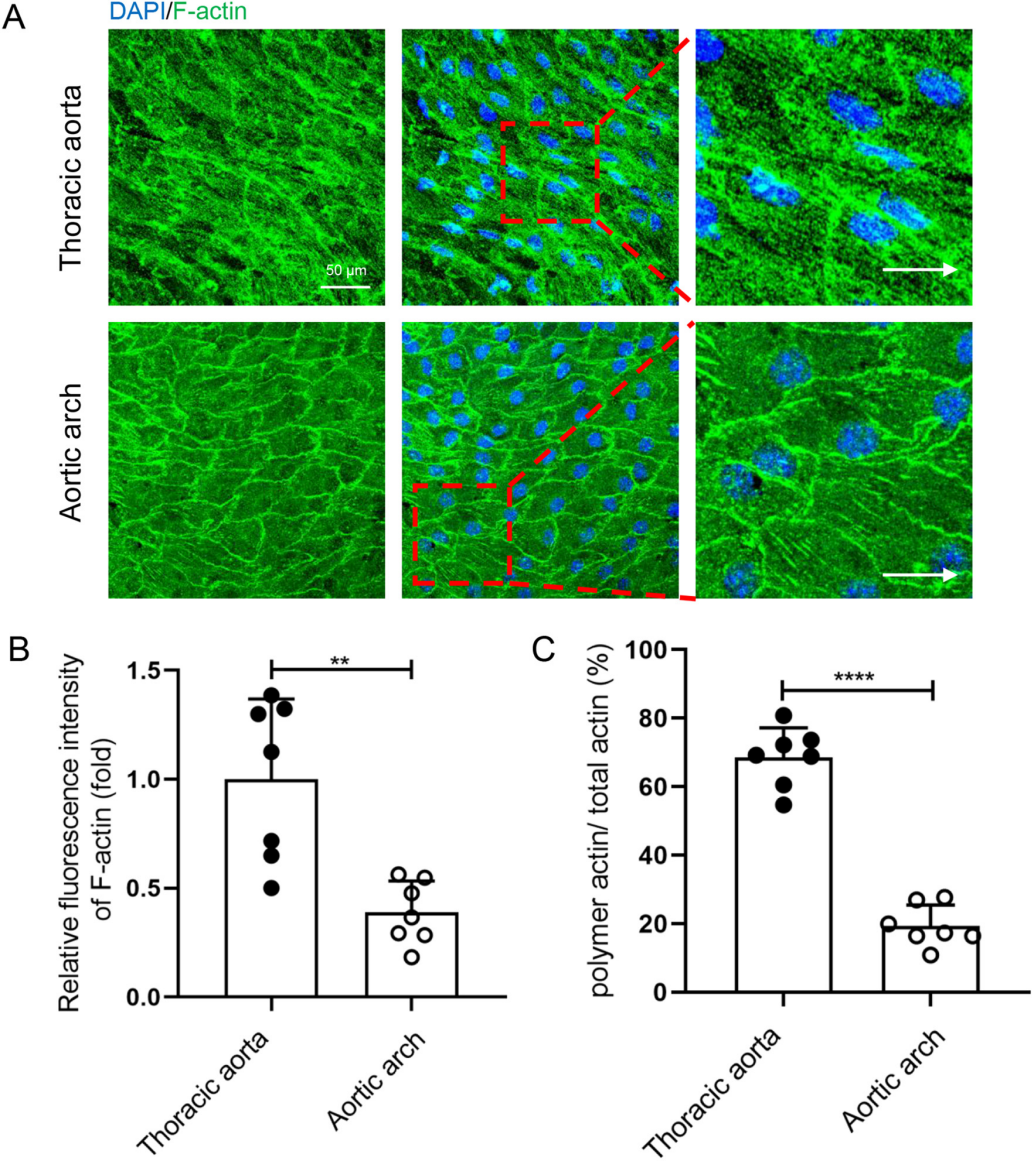
OSS inhibited the polymerization of F-actin *in vivo*. (a) Immunofluorescence staining and en face for F-actin (green) and nuclei (blue) in endothelial cells of thoracic aorta and aortic arch from C57BL/6J mice (WT mice). White arrows pointed in the direction of blood flow (scale bar = 50 *μ*m). (b) Statistical analysis of the relative fluorescence intensity as shown in (a) (n = 7). (c) Statistical analysis of the polymeric actin ratio (ratio of polymeric actin/total actin) as shown in (a) (n = 7). All data were presented as mean ± SD. ^**^ P < 0.01 and ^****^ P < 0.0001.

The distribution and arrangement of cytoskeleton (microtubules/MT and microfilaments/F-actin) is an important feature of PCP in ECs.^31,32^ To further investigate the effect of OSS on endothelial PCP, cell cytoskeleton in the inner curvature of the aortic arch and thoracic aorta was observed by immunofluorescence. In the endothelium of the thoracic aorta, microtubules emanating from the microtubule organizing center (MTOC) tend to be distributed preferentially in the upstream region along the cell long axis [Fig. S1(a)]. MTOC is redistributed around the nucleus, mainly on upstream and sides of the nuclear long axis, and rarely on downstream. Compared with ECs in thoracic aorta, MTOC on upstream of the long axis in ECs in inner curvature of aortic arch were significantly increased, on sides were significantly decreased, and on downstream had no difference [Fig. S1(c)]. F-actin, another major cytoskeleton, maintains cell shape and polarity of intracellular organization. Therefore, we examined the rearrangements of actin cytoskeleton. The results showed that thoracic aorta ECs contained a large number of F-actin, which aligned parallel to the cell long axis, and most of the F-actin across the cell. By contrast, aortic arch ECs showed a circular line of F-actin along the cell edge, and only a small amount of F-actin with shorter length was present in the cells [Fig. 2(a)]. We analyzed the fluorescence intensity of microfilaments and the percentage of polymeric actin (polymer actin area/total actin area) in ECs from the aortic arch and thoracic aorta and found that the fluorescence intensity of F-actin and proportion of polymeric actin of ECs in the inner curvature of aortic arch was significantly reduced compared with ECs in the thoracic aorta [Figs. 2(b) and 2(c)].

Golgi apparatus in polarized ECs induced by blood flow mainly located on upstream of the nuclear long axis is a sign of PCP.^32^ Intracellular localization of Golgi apparatus in ECs of inner curvature of the aortic arch and thoracic aorta were observed by immunofluorescence. As anticipated, Golgi apparatus were mainly located on upstream and sides of the nuclear long axis, and few Golgi apparatus were distributed on downstream in ECs of the thoracic aorta [Fig. S2(a)]. The Golgi apparatus on upstream in the ECs of inner curvature of the aortic arch were significantly decreased, while Golgi apparatus on sides were significantly increased [Fig. S2(c)].

Together, these results demonstrate that compared to LSS, OSS inhibited the PCP in vascular ECs *in vivo*.

## RESULT 2: OSS INHIBITED VASCULAR ENDOTHELIAL PLANAR CELL POLARITY *IN VITRO*

**FIG. 3.**
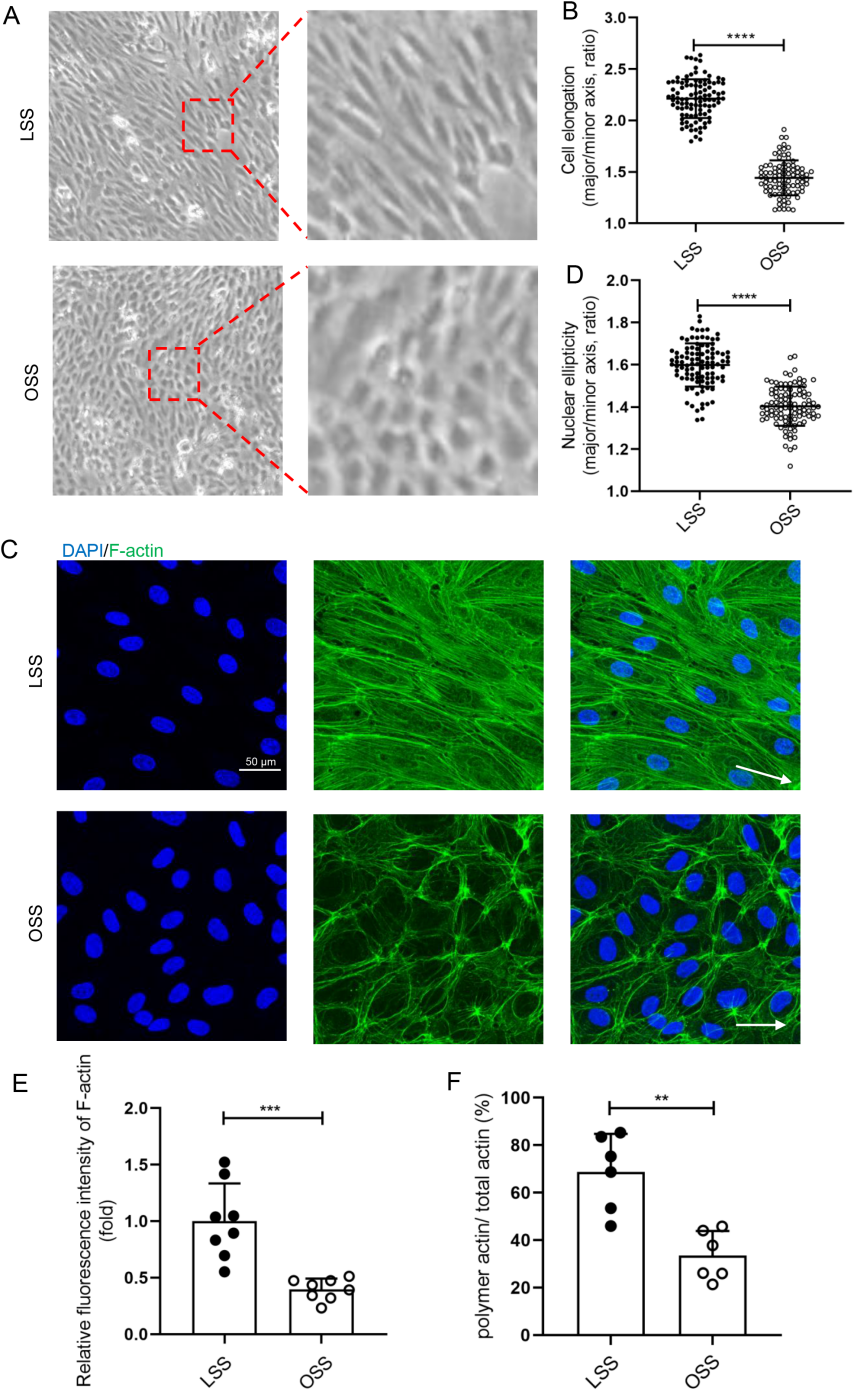
OSS inhibited the polymerization of F-actin *in vitro*. (a) and (c) HUVECs were loaded with LSS (12 dyn/cm^2^) and OSS (5 dyn/cm^2^) for 48 h. (a) Representative photomicrographs of HUVECs morphology after mechanical loading. White arrows pointed in the direction of loading fluid. (b) Statistical analysis of the cell elongation (ratio of long axis/short axis of cells) (n > 80). (c) Immunofluorescence staining for F-actin (green) and nuclei (blue) in HUVECs. White arrows pointed in the direction of loading fluid (scale bar = 50 *μ*m). (d) Statistical analysis of nuclear ellipticity (ratio of major axis/minor axis of nucleus) as shown in (c) (n = 100). (e) Statistical analysis of the relative fluorescence intensity of F-actin as shown in (c) (n = 8). (f) Statistical analysis of the polymeric actin ratio (ratio of polymer actin/total actin) as shown in (c) (n = 6). All data were presented as mean ± SD. ^**^ P < 0.01, ^***^ P < 0.001, and ^****^ P < 0.0001.

To further investigate the effect of OSS on endothelial PCP, HUVECs were exposed to LSS or OSS for 48 h through a parallel-plate flow chamber device *in vitro*. We observed morphological changes of ECs stimulated by different shear stress. LSS-stimulated HUVECs mainly exhibited a long spindle shape, and the cell long axis direction paralleled to the fluid direction [Fig. 3(a)]. The elongated HUVECs stimulated by LSS *in vitro* were more obvious than the thoracic aorta ECs. Compared to LSS, cell elongation was significantly inhibited under OSS conditions [Fig. 3(b)].

*In vitro*, we examined the effects of different shear stress on HUVEC cytoskeleton including microtubules and microfilaments. Unlike *in vivo* EC localization, MTOC was mainly distributed on sides of the nuclear long axis, while the proportion of MTOC on the upstream was few [Figs. S3(a) and S3(c)]. Compared with LSS-stimulated HUVECs, OSS significantly increased the distribution of MTOC upstream of the long axis of the nucleus but decreased the distribution on both sides of the nucleus [Fig. S3(c)]. The pattern of microfilaments *in vitro* was similar to that in *in vivo*. LSS-stimulated HUVECs contained a large number of F-actin, which aligned parallel to the long axis of the cell, and most of the F-actin also across the cell. By contrast, OSS-stimulated HUVECs exhibited a circular band of F-actin along the cell edge, and a small amount of F-actin with a shorter length also presented in the cells [Fig. 3(c)]. We calculated the fluorescence intensity of the microfilaments and the percentage of polymerization actin of HUVECs stimulated by LSS or OSS and found that OSS reduced the fluorescence intensity of F-actin and the percentage of polymerization actin [Figs. 3(d) and 3(f)].

*In vitro*, the Golgi apparatus was more likely to distribute on sides of the long axis of the nucleus in HUVECs exposed to OSS compared to LSS (Fig. S4). This phenomenon is more pronounced in *in vitro* than in *in vivo*. In other words, OSS inhibited vascular endothelial PCP in *in vivo* and *in vitro*.

## RESULT 3: OVEREXPRESSED TET1s RESCUED OSS-INDUCED ENDOTHELIAL PCP DAMAGE

To investigate the effect of different shear stress on TET1s expression in ECs, HUVECs were exposed to LSS or OSS for 48 h using a parallel plate flow chamber mechanical loading device. Immunofluorescence and western blot results exhibited that TET1s expression levels in OSS group were significantly reduced compared to LSS in HUVECs (Fig. S5). These results suggest that OSS inhibited TET1s expression in ECs.

**FIG. 4.**
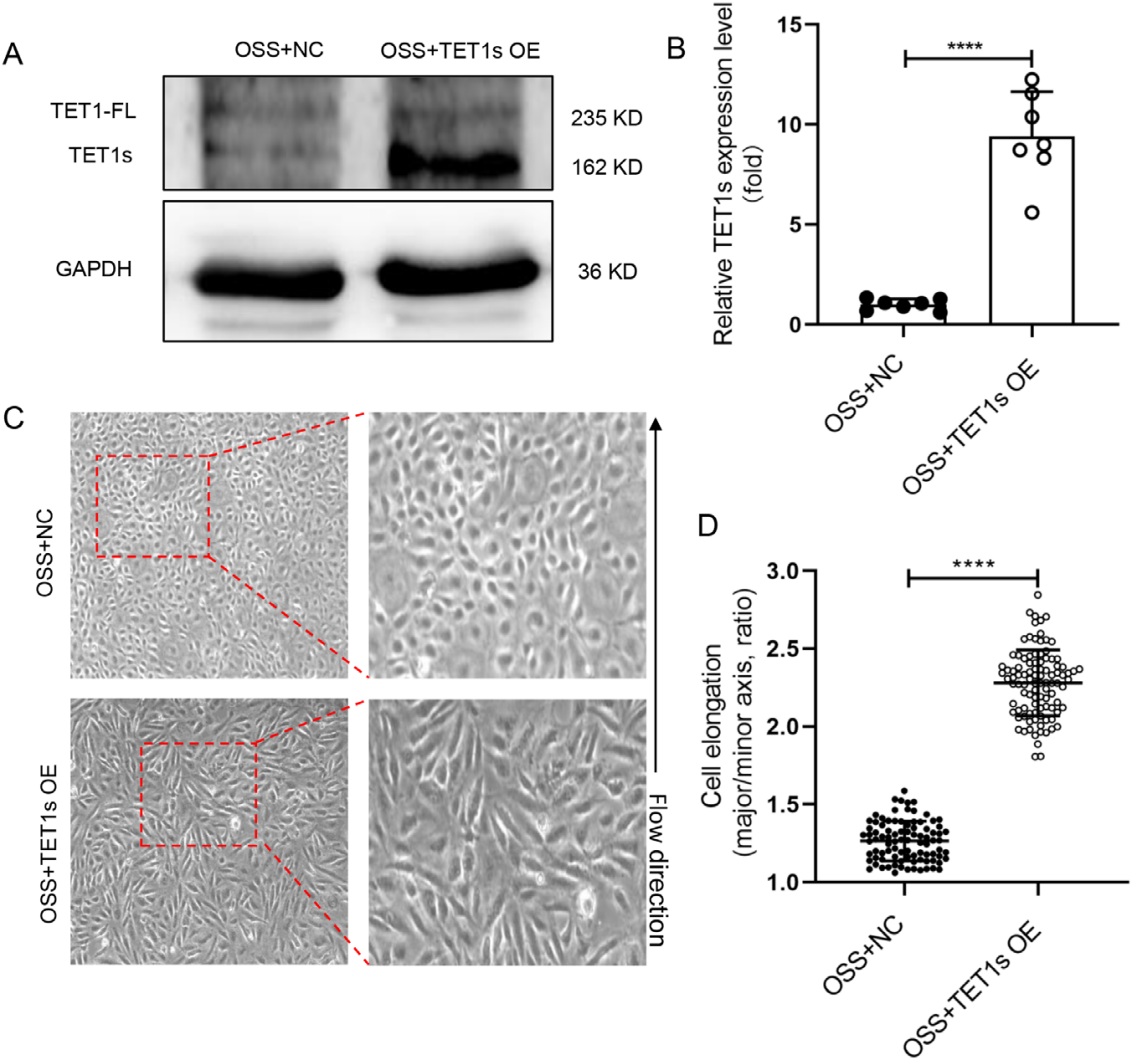
Overexpressed TET1s rescued OSS-inhibited endothelial PCP. (a) and (b) HUVECs were transfected with TET1-overexpressed adenovirus or control adenovirus and loaded with OSS for 48 h. Western blot was performed to detect the expression levels of TET1-FL and TET1s proteins in HUVECs. (c) Representative photomicrographs of morphology of TET1s-overexpressed HUVECs. (d) Statistical analysis of the elongation (the ratio of cellular major axis/minor axis) as shown in (a) (n > 90) and as shown in (c). All data were presented as mean ± SD. ^****^ P < 0.0001.

**FIG. 5.**
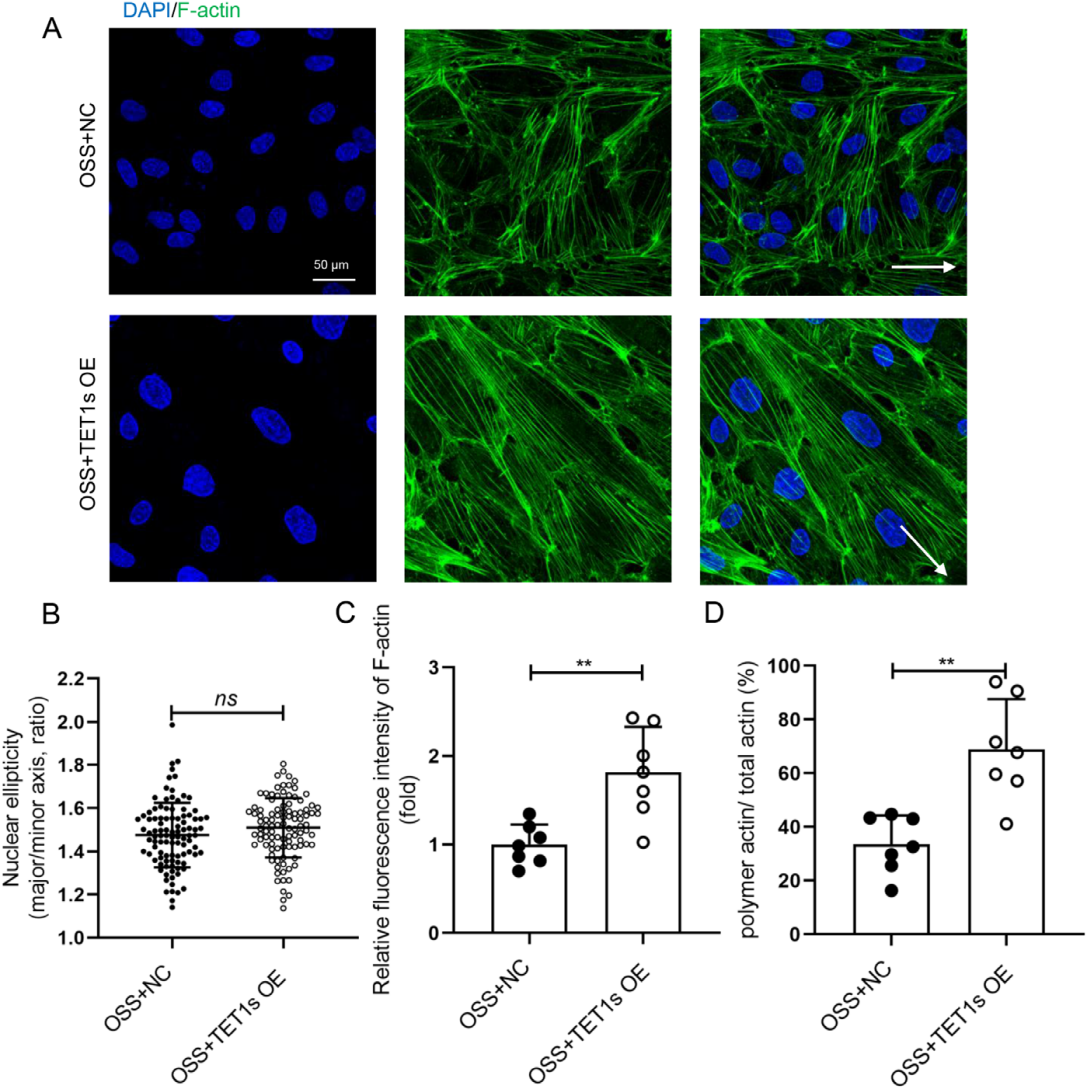
Overexpressed TET1s alleviated OSS-suppressed endothelial F-actin polymerization. (a) HUVECs were transfected with TET1-overexpressed adenovirus or control adenovirus and loaded with OSS for 48 h. Immunofluorescence staining for F-actin (green) and nuclei (blue) in HUVECs. (b) Statistical analysis of nuclear ellipticity (ratio of major axis/minor axis of nucleus) as shown in (a) (n = 100). (c) Statistical analysis of the relative fluorescence intensity of actin as shown in (a) (n = 7). (d) Statistical analysis of polymetric actin ratio (ratio of polymer actin/total actin) as shown in (a) (n = 7). All data were presented as mean ± SD. ns means no significant difference; ^**^ P < 0.01.

To examine whether the decrease in TET1s is the key factor in OSS-induced endothelial PCP damage, we constructed an TET1s-overexpressed adenovirus to transfect HUVECs, which were also exposed to OSS [Figs. 4(a) and 4(b)]. Compared with the negative control (NC) group, the cell elongation in the overexpressed TET1s (OE) group was significantly increased [Figs. 4(c) and 4(d)]. However, there was no significant change in nuclear ellipticity [Fig. 5(b)], which may be regulated by flow-related other pathways, not TET1s.

We also tested the effect of TET1s overexpression on the localization of endothelial cytoskeleton and Golgi apparatus in HUVECs exposed to OSS. In TET1s-overexpressed HUVECs, the number and length of F-actin were significantly increased, and most of the F-actin paralleled to the direction of blood flow [Fig. 5(a)]. Compared with the NC group, actin fluorescence intensity and polymerization actin ratio of OE group was significantly increased [Figs. 5(c) and 5(d)]. Finally, we analyzed the localization of Golgi apparatus relative to the nucleus, and found that the localization of MTOC (Fig. S6) and Golgi apparatus (Figs. S6, S7) relative to the nucleus in TET1s-overexpressed HUVECs had no difference. These data indicate that TET1s overexpression may rescue the endothelial PCP inhibited by OSS.

## RESULT 4: DELETION OF TET1s DAMAGED LSS-INDUCED ENDOTHELIAL PCP

**FIG. 6.**
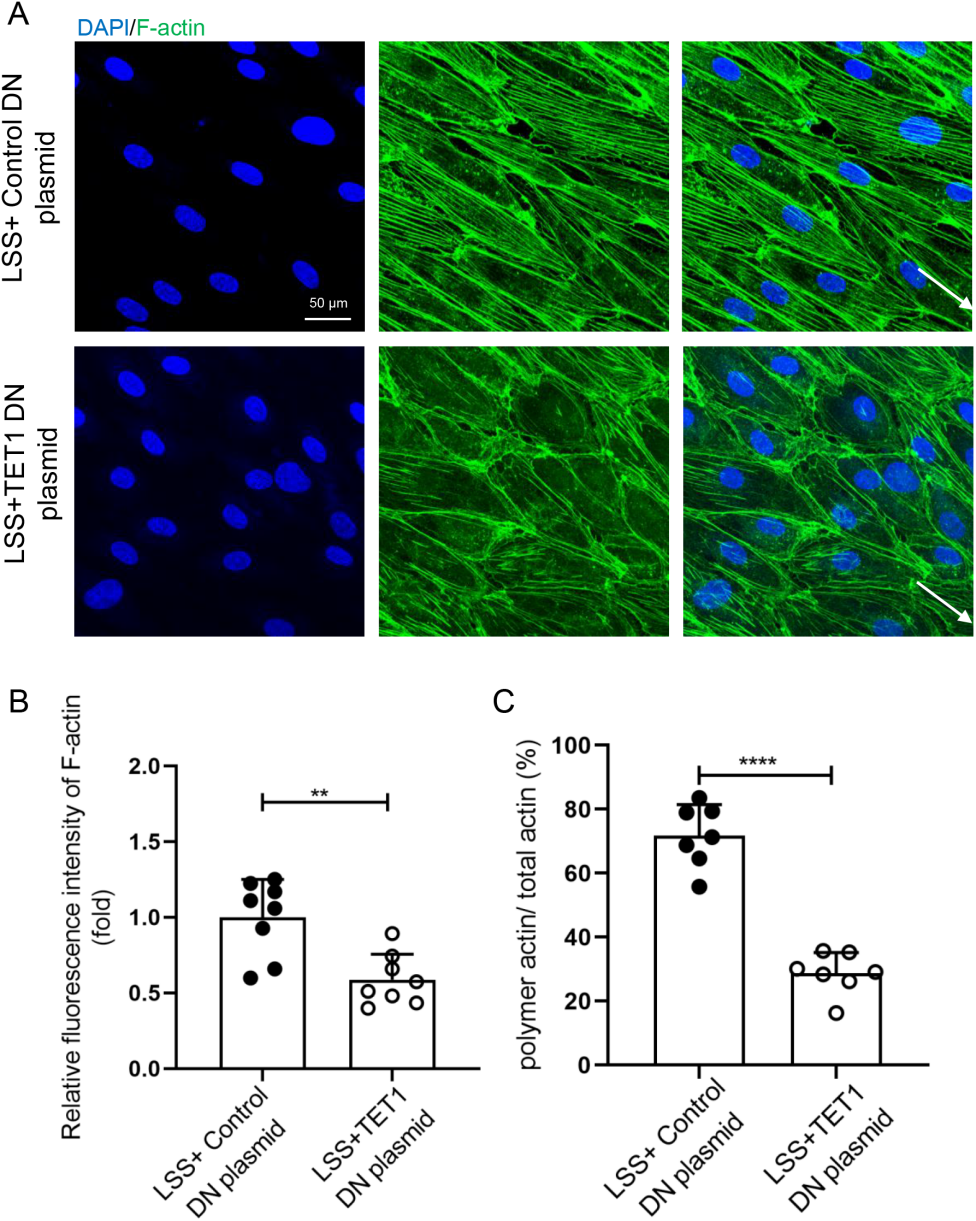
Knockout TET1s inhibited LSS-induced polymerization of endothelial F-actin *in vitro*. (a) HUVECs were transfected with TET1 double nickase plasmid to block the expression of TET1s or control plasmid and loaded with LSS (12 dyn/cm^2^) for 48 h. Immunofluorescence staining for F-actin (green) and nuclei (blue) in HUVECs. (b) Statistical analysis of the relative fluorescence intensity of actin as shown in (a) (n = 7). (c) Statistical analysis of polymetric actin ratio (ratio of polymer actin/total actin) as shown in (a) (n = 7). All data were presented as mean ± SD. ^**^ P < 0.01 and ^****^ P < 0.0001.

The above results showed that overexpression of TET1s partially restored OSS-induced endothelial PCP damage through enhancing intracellular F-actin polymerization, increasing the number and length of F-actin, and regulating the F-actin alignment with the flow direction. However, the localization and distribution of endothelial microtubules and Golgi apparatus are not observably affected. Hence, we focus on the effect of TET1s on the microfilament. *In vitro*, we knocked out the expression of TET1s in HUVECs using a TET1 knockout plasmid, and at the same time, loaded LSS for 48 h. The results showed that compared with the mock-vehicle group, knocking out the expression of TET1s decreased the number and length of LSS-induced F-actin polymerization in HUVECs, and the alignment of F-actin paralleled to the flow direction was also significantly reduced [Fig. 6(a)]. Meanwhile, fluorescence intensity and polymerization ratio of F-actin were also decreased [Figs. 6(b) and 6(c)].

TET1^−/−^ and TET1^cs/cs^ mice were used to further investigate the effect of TET1s on endothelial microfilament *in vivo*, the only difference being whether they expressed TET1s or not. By comparing the difference in the thoracic aorta ECs between TET1^−/−^ and TET1^cs/cs^ mice, we found that the deficiency of TET1s reduced the polymerization ratio and length of F-actin (Fig. 6).

## RESULT 5: sFRP-1/Fzd4 MEDIATED TET1s-INDUCED F-ACTIN POLYMERIZATION

**FIG. 7.**
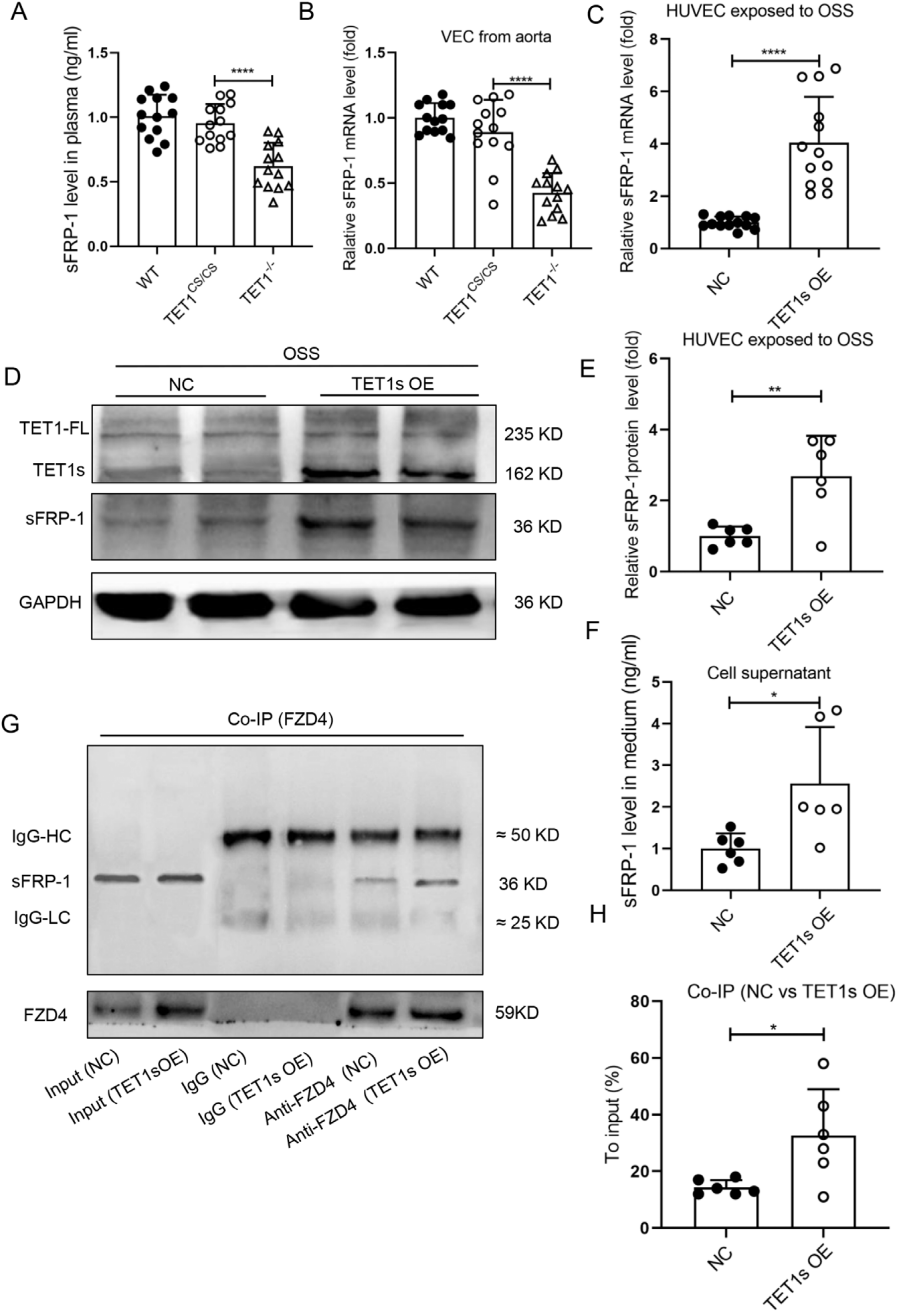
Overexpression of TET1s promoted sFRP-1 expression in endothelial cells. (a) Enzyme-linked immunosorbent assay (ELISA) was used to detect the level of sFRP-1 in plasma of WT mice, TET1cs/cs mice, and TET1^−/−^ mice (n = 13). (b) Quantitative PCR detected the relative mRNA levels of sFRP-1 in ECs from the aorta of WT mice, TET1^cs/cs^ mice, and TET1^−/−^ mice (n = 13). (c)–(g) HUVECs were transfected with TET1-overexpressed adenovirus or control adenovirus and loaded with OSS for 48 h. (c) Quantitative PCR was performed to detect the relative mRNA level of sFRP-1. (d) and (e) Western blotting was performed to detect the protein expression levels of TET1-FL, TET1s, and sFRP-1 (n = 6). (f) ELISA was used to detect the level of sFRP-1 in the medium (n = 6). (g) and (h) Co-IP was used to detect the interaction of Fzd4 with sFRP-1 (n = 4). All data were presented as mean ± SD. ^*^ P < 0.05, ^**^ P < 0.01, and ^****^ P < 0.0001.

Secreted frizzled-related protein-1 (sFRP-1) regulates the EC cytoskeletal reorganization, the expression of which is regulated by epigenetic pathways.^33^ To further explore the mechanism of TET1s-involved endothelial PCP regulation, plasma sFRP-1 levels in TET1^−/−^ mice and TET1^cs/cs^ mice were measured by ELISA, and it was found that the deficiency of TET1s significantly reduced plasma sFRP-1 levels [Fig. 7(a)]. ECs were isolated from the thoracic aorta of TET1^−/−^ mice and TET1^cs/cs^ mice, and the mRNA expression level of sFRP-1 was detected by RT-qPCR. The deletion of TET1s obviously reduced the mRNA expression level of sFRP-1 [Fig. 7(b)]. On the contrary, *in vitro*, overexpressed-TET1s in HUVECs exposed to OSS increased the expression and secretion of sFRP-1 [Figs. 7(c)–7(f)].

ECs highly express frizzled protein 4 (Fzd4), a member of the Fzd family, and it has been confirmed that sFRP-1/Fzd4 regulates the cytoskeleton reorganization.^33^ By immunoprecipitation technique, we confirmed that OSS significantly decreased the sFRP-1/Fzd4 interaction (Fig. S8). However, overexpression of TET1s partially rescued the interaction of sFRP-1 with Fzd4 that is inhibited by OSS [Figs. 7(g) and 7(h)].

**FIG. 8.**
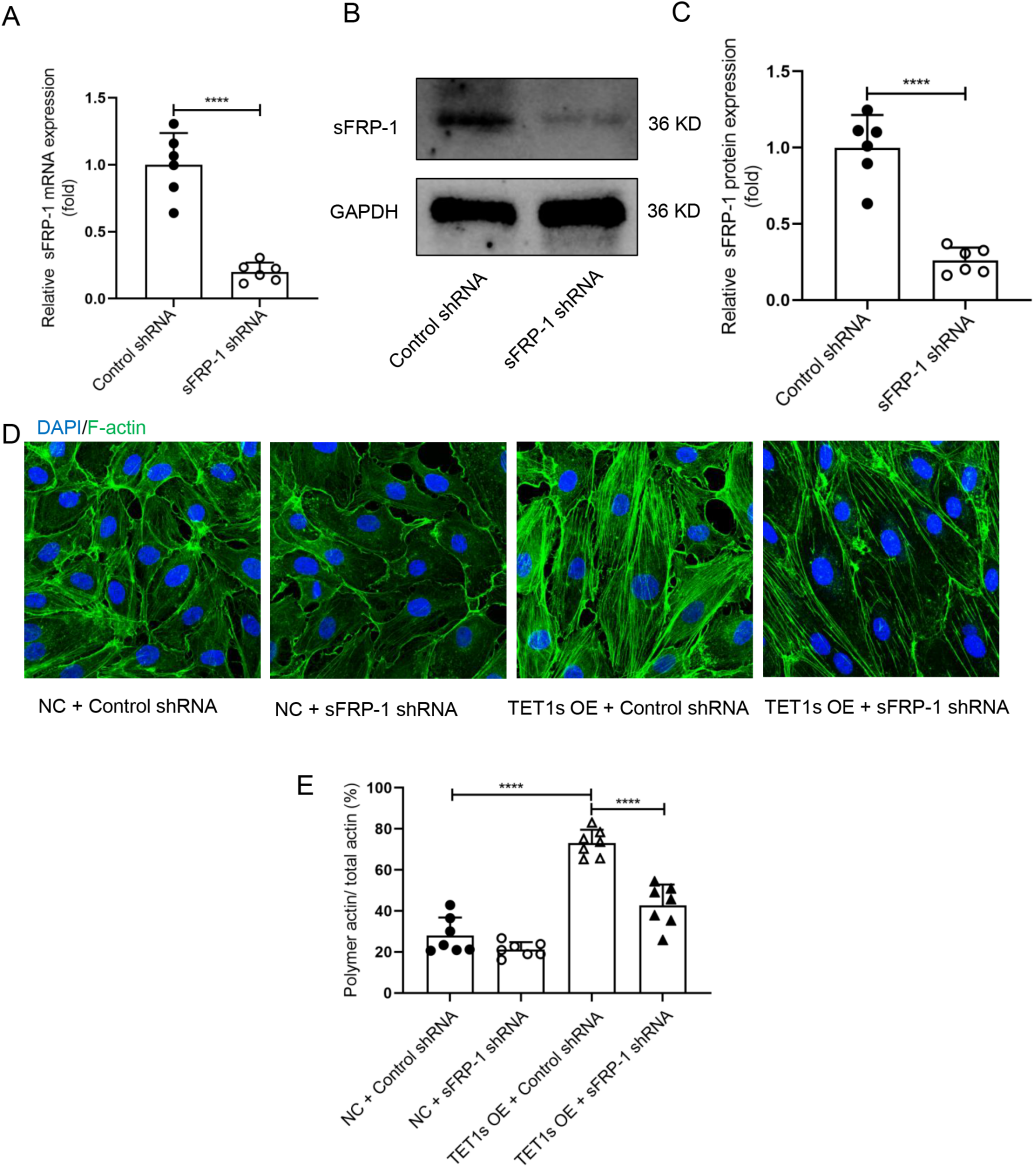
Knockdown sFRP-1 blocked overexpression TET1s-induced polymerization of endothelial F-actin. (a)–(c) HUVECs transfected sFRP-1 shRNA plasmid or control shRNA plasmids. (a) Quantitative PCR to detect the relative mRNA levels of sFRP-1 (n = 6). (b) and (c) Western blotting detected the protein level of sFRP-1 (n = 6). (d) HUVECs were transfected with TET1-overexpressed adenovirus or control adenovirus and transfected with sFRP-1 shRNA plasmid or control shRNA plasmids and loaded with OSS for 48 h. Immunofluorescence staining for F-actin (green) and nuclei (blue) in HUVECs. White arrows pointed in the direction of loading fluid (scale bar = 50 *μ*m). (e) Statistical analysis of polymetric actin ratio (ratio of polymer actin/total actin) as shown in (d) (n = 7). All data were presented as mean ± SD. ^****^ P < 0.0001.

To confirm that the sFRP-1/Fzd4 signaling pathway is involved in TET1s-induced F-actin polymerization, we knocked down sFRP-1 expression by transfecting sFRP-1 interfering plasmid into overexpressed-TET1s HUVECs [Figs. 8(a)–8(c)]. As shown in the representative images, knockdown sFRP-1 expression impaired the TET1s-induced polymerization of F-actin [Figs. 8(d) and 8(e)].

## RESULT 6: TET1s PROMOTED sFRP-1 EXPRESSION BY DEMETHYLATION

**FIG. 9.**
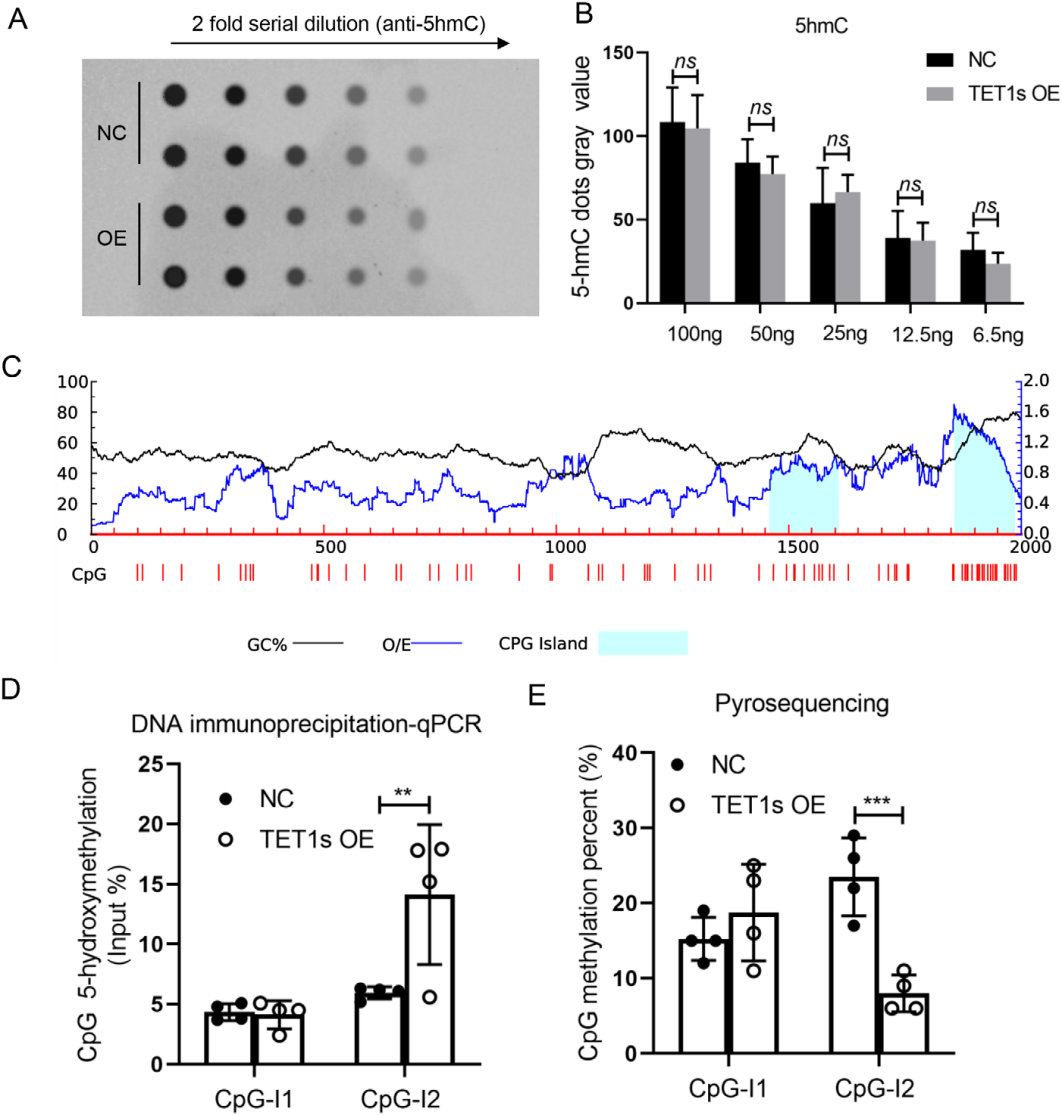
Overexpression of TET1s in HUVECs promoted sFRP-1 promoter demethylation. (a), (b), (d), and (e) HUVECs were transfected with TET1-overexpressed adenovirus or control adenovirus, and loaded with OSS for 48 h. (a) and (b) Dot-blot assay detected the level of global 5hmC in HUVECs (n = 4). (c) The distribution of CpG island sites within 2000 bp of the sFRP-1 promoter region was predicted by MethPrimer2.0. Island1, 160 bp in length, located at 1454-1613; Island2, 115 bp in length, located at 1831-1945. (d) DNA immunoprecipitation-qPCR tested the levels of 5 mC in two CpG island (n = 4). (e) Pyrosequencing assay analyzed the local 5hmC levels in two CpG island. All data were presented as mean ± SD. ns means no significant difference; ^**^ P < 0.01 and ^***^ P < 0.001.

We have demonstrated that TET1s promotes the expression of sFRP-1 in ECs, but the mechanism remains unclear. The function of TET family is to mediate DNA demethylation and promote gene transcription. We analyzed the sFRP-1 gene promoter by MethPrimer and found that there were two distinct CpG islands in the upstream 2000 bp of the sFRP-1 gene promoter [Fig. 9(c)]. To investigate whether TET1s promotes the expression of sFRP-1 in ECs through DNA demethylation, we first detected the global DNA methylation (5mC) and hydroxymethylation (5hmC) levels of overexpressed-TET1s HUVECs exposed to OSS by dot-blot experiment. The results indicated that overexpressed-TET1s did not lead to the changes in global DNA methylated (Fig. S9) and hydroxymethylated levels [Fig. 9(a)] in HUVECs. DNA immunoprecipitation-qPCR was used to analyze the hydroxymethylation level of CpG island, and the results exhibited that overexpressed-TET1s increased the hydroxymethylation level of the second CpG island of the sFRP-1 gene promoter [Fig. 9(d)]. In addition, we confirmed that TET1s significantly reduced the methylation level of the second CpG island of the sFRP-1 gene promoter by pyrosequencing [Fig. 9(e)]. These results suggest that overexpression of TET1s mediates the demethylation of the sFRP-1 gene promoter.

Hence, these results demonstrate that TET1s is a key factor in the regulation of FSS-induced endothelial PCP. This regulation is by altering the demethylation of sFRP-1 promoter, thereby affecting the interaction of sFRP-1/Fzd4, and ultimately mediating F-actin.

## DISCUSSION

There are different types of hemodynamics in the body.^34^ Although FSS can be classified in various ways, most researchers divide flow shear stress into OSS, which promotes vascular disturbance, and LSS, which maintains vascular stability.^35^ It has been demonstrated that the FSS induces endothelial PCP change.^16^ Here, we demonstrated that compared to LSS, OSS significantly inhibits endothelial PCP both *in vivo* and *in vitro* (Figs. 1–3 and S1–S5). Several studies demonstrated that the stiff substrate saturates the effect of FSS on endothelial polarity.^36^ In our study, more F-action formation was observed in ECs cultured on glass plates (a stiffer substrate than that used in *in vivo*) in a parallel plate flow chamber device *in vitro*. It may present the intrinsic limitation in term of decoupling the effects of substrate stiffness in studying the effects of FSS in the study.

The cellular morphological change is the most direct change of cell polarization.^16^ The nucleus is the largest organelle in a cell, and its long axis orientation is often the same as that of the cell.^37,38^ The nucleus is also the blood flow sensor of the cell, and its orientation is regulated by blood flow. Endothelial nucleus creates a bulge on the apical cell surface. Hence, hydrodynamic stresses around the cell may mechanically push this bulge downstream, inducing planar polarization of ECs.^11,39^ The cytoskeleton is a key step in the formation and maintenance of PCP in ECs.^40,41^ Microtubules, one of the cytoskeletons, serve as guiding structures for polarized cytoplasm and organelles and also serve as vector and selective transport of intracellular vesicles. MTOC radiates around and controls the formation and distribution of microtubules.^42^ Microfilaments of ECs are mainly composed of actin; the monomer actin is called G-actin, and the polymerized actin is called F-actin, namely, microfilaments. Actin polymerizes from G-actin to form F-actin, which helps maintain cell shape and polarity.^43,44^ Intracellular localization of Golgi apparatus is also a marker of changes in endothelial cell polarization.^45^ Here, we selected morphological indexes including nuclear ellipticity, cytoskeleton organization, and Golgi apparatus localization to assess the PCP difference in ECs.

It was reported that EC polarization against the flow was stronger in high flow arteries than in veins. In our study, HUVECs were used *in vitro* test. Though the HUVEC is a commonly used model for *in vitro* evaluation to study physiological and pathological responses of vascular endothelium, it may still present an indeterminate limitation in studying of the endothelial polarization in response FSS.

Ten-eleven translocation (TET) proteins, including TET1, TET2, TET3, could regulate gene expression by mediating DNA demethylation. All these three TET proteins can oxidize 5-methylcytosine (5mC) to 5-hydroxymethylcytosine (5hmC), which can be further processed into cytosine through base excision repair, leading to DNA demethylation ultimately.^46^ As a truncated TET1, TET1s still shares the catalytic activity with TET1-FL to oxidize 5mC to 5hmC.^21^ However, TET1-FL leads to the upregulation (by 7.5-fold) of more genes than TET1s.^22^ In this study, overexpressed TET1s in HUVECs did not lead to the changes in global DNA methylated and hydroxymethylated levels, while it increased the hydroxymethylated levels of sFRP-1 promoter and increased its expression level [Figs. 9(a), 9(d), 9(e), and S9]. This showed that TET1s can achieve the dynamic balanced control of the methylated and hydroxymethylated levels.

**FIG. 10.**
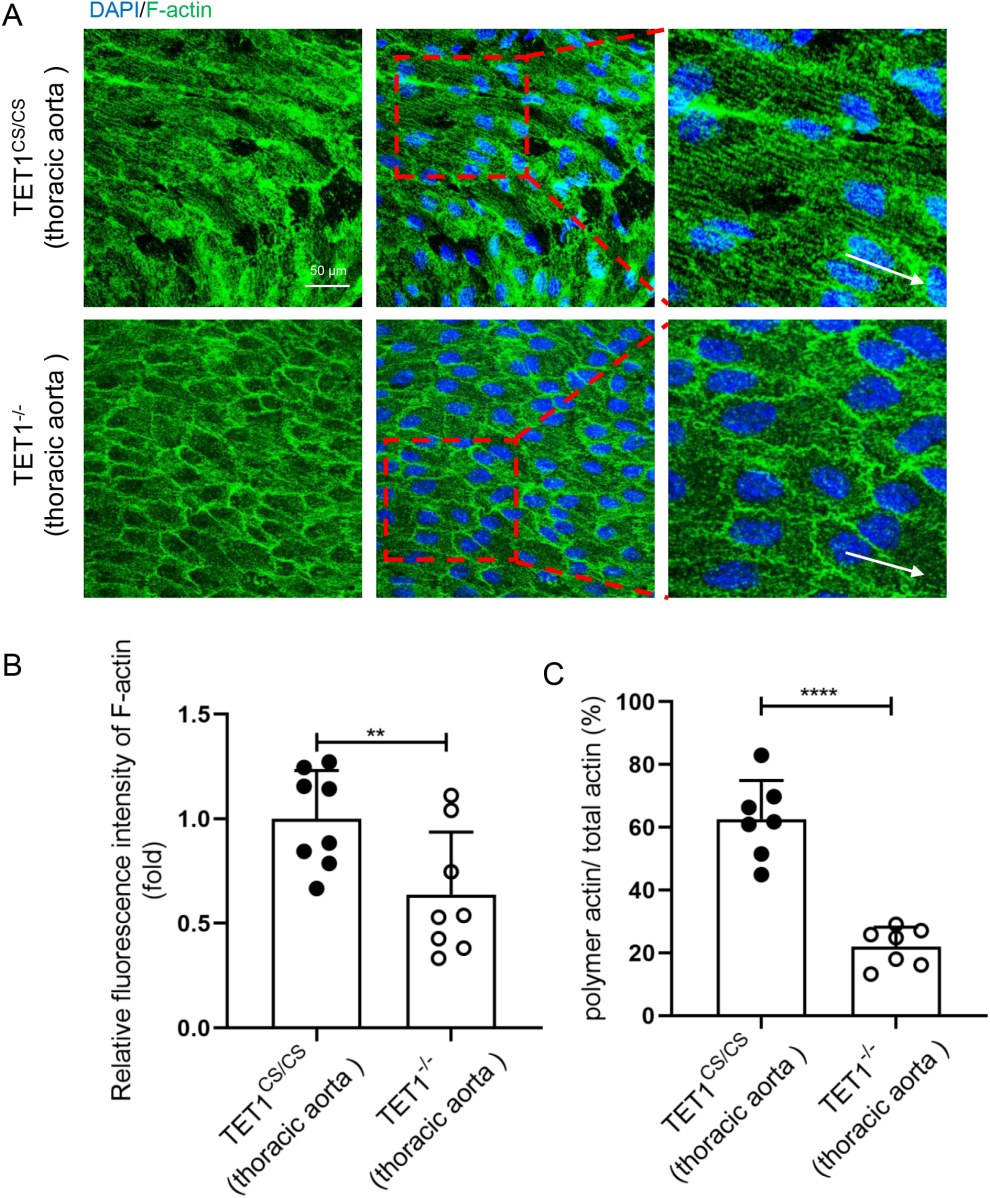
Loss of TET1s inhibited LSS-induced polymerization of endothelial F-actin *in vivo*. (a) Immunofluorescence staining and en face for F-actin (green) and nuclei (blue) in endothelial cells of thoracic aorta from TET1^cs/cs^ and TET1^−/−^ mice. White arrows pointed in the direction of blood flow (sbar = 50 *μ*m). (b) Statistical analysis of the relative fluorescence intensity of actin as shown in (a) (n = 7). (c) Statistical analysis of polymetric actin ratio (ratio of polymer actin/total actin) as shown in (a) (n = 7). All data were presented as mean ± SD. ^**^ P < 0.01 and ^****^ P < 0.0001.

TET1s expression in ECs is a predominant transcript compared with TET1-FL.^23^ Our previous studies have shown that TET1s is mechanosensitive, LSS promotes TET1s expression, while OSS inhibits TET1s expression, and the expression of TET1s is involved in protecting the vascular endothelial barrier.^23^ Here, we showed that overexpressed-TET1s in HUVECs exposed to OSS can partially restore endothelial PCP by enhancing the polymerization of actin (Figs. 4 and 5). Deletion of TET1s in HUVECs exposed to LSS significantly impaired actin polymerization (Figs. 6 and 10). These results suggest that OSS inhibits endothelial PCP, partially due to the inhibition of TET1s expression.

Because the TET1s gene shares a vast majority of exon with TET1-FL, it is difficult for us to solely knockout the TET1s gene. To further research the effect of TET1s on EC polarization *in vivo*, TET1^−/−^ mice without TET1-FL and TET1s, and TET1^cs/cs^ mice without TET-FL were introduced. Both types of mice are deficient in TET1-FL, and the only difference between them lies in the expression of TET1s. *In vivo*, deletion of TET1s significantly impaired actin polymerization in ECs. However, we did not apply a specific endothelial knockout model in mice. TET1s is not only expressed in ECs but also expressed in other cells. The effect of TET1s on ECs actin polymerization cannot be completely attributed to endothelial TET1s. Fortunately, *in vitro*, overexpressed TET1s in ECs partly verifies the positive effect of TET1s on EC actin polymerization.

Fzd4 is a membrane receptor of Wnt signaling pathway, which plays an important role in regulating PCP.^47^ Studies have shown that Fzd4 interacting with sFRP-1 increases the spread area of ECs on the extracellular matrix by promoting the reorganization of endothelial cytoskeleton F-actin.^33^ Our results confirmed that overexpression of TET1s in ECs promoted the expression and secretion of sFRP-1 [Figs. 7(a)–7(f)]. In addition, the interaction between sFRP-1 and Fzd4 was significantly enhanced under the condition of overexpression of TET1s or LSS-induced high expression of TET1s [Figs. 7(g), 7(h) and S8]. By interfering with the expression of sFRP-1 in ECs, TET1s overexpression-induced polymerization of actin was effectively inhibited [Figs. 8(d) and 8(e)]. These results suggest that sFRP-1 is a key factor in the regulation of TET1s-induced F-actin polymerization.

Taken together, this study demonstrates that TET1s is involved in the regulation of FSS-induced endothelial PCP change. TET1s promotes sFRP-1 demethylation level in the sFRP-1 promoter region. As a regulatory factor of EC cytoskeletal reorganization, the high expression of sFRP-1 leads to F-actin polymerization through a sFRP-1/Fzd4 signal pathway, thus inducing endothelial PCP. These findings not only extend our understanding of the role of TET1s in OSS-induced PCP damage but could also be exploited for the potential treatment of OSS-driven vascular disease.

## METHODS

### Animals

TET1^−/−^ mice were obtained from Chen Dong's group at Tsinghua University. TET1^cs/cs^ mice were obtained from Wei Xie's group at Tsinghua University. Wild-type C57BL/6J mice were purchased from Beijing Vital River Laboratory Animal Technology Co., Ltd. TET1^−/−^ mice and TET1^cs/cs^ mice have been described in detail in previous studies.^22,25^ Briefly, TET1^−/−^ mice neither express TET1 full-length (TET1-FL) nor TET1s, and TET1^cs/cs^ mice do not express TET1-FL, but only TET1s.

### Cell culture

Primary human umbilical vein endothelial cells (HUVECs) were purchased from ScienCell Company. Before subculture, cellular vessels were coated with plasma fibronectin (8018, ScienCell) (40 *μ*g/ml) for 12 h. HUVECs were cultured in EC medium (ECM, ScienCell) supplemented with 5% fetal calf serum (0025, ScienCell), 1% penicillin/streptomycin (0503, ScienCell), and endothelial growth supplement (1052, ScienCell). All cells were cultured at 37 °C in a 5% CO_2_ atmosphere and tested negative for mycoplasma.

### Plasmid transfection

TET1 CRISPR/Cas9 KO (sc-400845-KO-2, Santa Cruz Biotechnology) plasmid and sFRP-1 shRNA plasmid (sc-39998-SH, Santa Cruz Biotechnology) were purchased from Santa Cruz Biotechnology. HUVECs were transfected at 60%–70% confluence with TET1 CRISPR/Cas9 KO plasmid or sFRP-1 shRNA plasmid using UltraCruz® Transfection Reagent (sc-395739, Santa Cruz Biotechnology) according to the manufacturer's protocols. HUVECs transfected with TET1 CRISPR/Cas9 KO plasmid or sFRP-1 shRNA plasmid were select by adding puromycin.

### Adenovirus constructs and transfection

The transcript sequence of TET1s was cloned into the pAV [Exp]-CMV>EGFP vector. Adenovirus particles were generated by transfection of HEK293A cells. Supernatants were collected after 72 h of transfection and concentrated by centrifugation (20 000 *×* *g*, 4 °C, 2 h). HUVECs were treated with TET1s overexpression adenovirus or empty vector adenovirus (as a negative control), and the transfection medium was changed 12 h post-transfection. Experiments were performed after 48 h of virus transfection.

### Isolation of ECs in mice

Aortas were obtained from C57BL/6J mice, TET1^−/−^ mice, or TET1^cs/cs^ mice. The anesthetized mice were perfused with PBS containing 1000 U/ml of heparin to wash the blood vessel. The adhering fat and tissue on the aorta were excised and then immersed in DMEM (ScienCell) containing 20% FBS and 1000 U/ml heparin. The whole aorta in the aortic arch and thoracic aorta were cut. The adventitia from the aortic arch and thoracic aorta were, respectively, separated. Adventitia-free aortas were immersed in PBS containing 0.25% of trypsin at 37 °C for 5 min. Then, 10% FBS was added to it to terminate digestion. The ECs were separated from the inner curvature area of the aortic arch and thoracic aorta by washing the intima with PBS. ECs were collected by centrifugation. The proportion of ECs were tested by flow cytometry.

### RNA isolation and RT-qPCR

Total RNA was extracted from the differently treated HUVECs or isolated ECs from the aorta using RNAiso Plus (#9109, Takara Biomedical Technology). The reverse transcription PCR Reagent Kit (RR047A, Takara Biomedical Technology) was used to reverse the transcription of RNA. Then, the relative mRNA levels were evaluated by the real-time quantitative PCR (RT-qPCR) system (CFX Connect^TM^, Bio-Rad). The following primers were used:

H-sFRP-1F: TGTGTCCTCCCTGTGACAAC
H-sFRP-1R: GTCAGCCCCATTCTTCAGGT
M-sFRP-1F: AAGCGAGTTTGCACTGAGGA
M-sFRP-1R: TACTGGCTCTTCACCTTGCG

### Parallel-plate flow chamber loading

A parallel-plate flow chamber was used to load shear stress in HUVECs. The device has been described in detail in Refs. 26 and 27. HUVECs were exposed to laminar flow (12 dyn/cm^2^) for 48 h, or low and oscillatory flow (5 dyn/cm^2^) for 48 h. Contrary to the laminar flow condition, the oscillatory flow apparatus contained a piston pump with a frequency of 1 Hz.

### Western blot

Total protein was extracted from the differently treated HUVECs or isolated ECs from the aorta using RIPA buffer containing PMSF (RIPA:PMSF = 96:4) (P0013, Beyotime). Protein concentrations were determined using a BCA Protein Assay Kit (P0010, Beyotime Biotechnology). Total protein samples were separated by SDS-PAGE (SK6010, Coolaber) and transferred onto a PVDF membrane (IPVH00010, Millipore) and blocked with skim milk for 2–4 h, and then washed with 1 *×* TBST, incubated with the primary antibody (TET1 antibody, GTX124207, GeneTex; GAPDH antibody, 10494-1-AP, Proteintech; sFRP-1 antibody, 26460-1-AP, Proteintech; Frizzled-4 antibody, sc-293454, Santa Cruz Biotechnology.) at 4 °C overnight. The membrane was washed and then incubated with a secondary antibody (A0208, Beyotime; A0216, Beyotime) at room temperature for 2 h. Finally, the membrane was washed again and visualized by ECL (P0018FM, Beyotime) in a gel imaging system.

### Cell immunofluorescence

Different treated HUVECs were fixed with 4% paraformaldehyde for 30 min. Then, the samples were washed with PBS and permeabilized/blocked with 0.1% Triton X-100 (in 5% BSA). Subsequently, the samples were incubated with the primary antibody (TET1 antibody, GTX124207, GeneTex; CD31 antibody, sc-376764, Santa Cruz Biotechnology; β-actin antibody, 8457, CST; GM130 antibody, sc-55591, Santa Cruz Biotechnology; CD144 antibody, 550548, BD Biosciences; γ-tubulin, 66320-1-Ig, Proteintech) in a wet box at 4 °C overnight. After the samples were washed with PBST (PBS with 0.1% Tween-20) for five times, the samples were incubated with secondary antibodies (ab150077, Abcam; ab150113, Abcam; 150078, Abcam) for 1 h in the dark. Nuclei were labeled with DAPI (D3571, Invitrogen) in the dark. The fluorescent signal was detected by SP8 confocal microscopy (Leica). Immunofluorescence intensity was analyzed by ImageJ.

### En face

Aortas were fixed with fresh 4% paraformaldehyde for 2 h, and then blocked and permeabilized with 5% BSA containing 1% Triton X-100 for 1 h. The diluted primary antibody was incubated overnight at a temperature of 4 °C. Following the primary antibody incubation, the samples were carefully washed to remove any unbound primary antibody. Subsequently, a secondary antibody (ab150077, Abcam; 150078, Abcam) was applied to the samples and incubated in a dark environment for 2 hours. After the secondary antibody incubation, another round of thorough washing was performed to remove any unbound secondary antibody. Finally, the samples were mounted with anti-fluorescence quenching mounting solution (containing DAPI) and photographed by SP8 confocal microscope. Immunofluorescence intensity was analyzed by ImageJ.

### Enzyme-linked immunoassay (ELISA)

Plasma sFRP-1 levels were measured by a manual ELISA. The sFRP-1 (26460-1-AP, Proteintech) antibody was diluted with coating buffer (0.05 mol/l Na_2_CO_3_-NaHCO_3_ Buffer, pH 9.6) to the desired concentration. Then, 200 μl of the diluted antibody was added to each well of the plate. The plate was incubated overnight at 4 °C to allow for specific binding of the antibody to its target. A blank control was included in the experiment as a negative control. The ELISA reaction plate was prepared. Then 200 μl of the antigen-containing sample, previously diluted in PBS, was added to each well of the plate. The plate was incubated at a temperature of 37 °C for 1 hour. The coating solution was removed and washed three times with PBST for 5 min each time; and to it was added 200 *μ*l of second HRP-labeled antibody solution diluted with PBS, and incubated at 37 °C for 60 min. Then, 200 *μ*l of tetramethylbenzidine (TMB) substrate solution was added in each well for 30 min at room temperature, and the reaction was stopped the reaction. The results were observed and recorded, and the OD value was measured with an enzyme label colorimeter (OPD = 492 nm), and the standard curve was used to convert the concentration.

### Immunoprecipitation

Immunoprecipitation has been described previously.^28^ In brief, HUVECs were lysed using Cell Lysis Buffer for western blotting or IP (P0013, Beyotime Biotechnology) for 15 min on ice. The cell lysate was incubated with anti-Fzd4 antibody for 2 h at 4 °C. Then, 50 *μ*l Protein A/G magnetic beads (HY-K0202, MCE) were added to the cell lysate sample containing the antigen for 2 h at 4 °C. The beads were washed with wash buffer (25 mM Tris-HCl pH 7.5, 100 mM NaCl, 10 mM MgCl_2_), and elution buffer was used to elute the target antigen. The final solution was used as a sample for denaturing SDS-PAGE.

### Dot-blot assay

The dot-blot assay has been described previously.^23^ Genomic DNA was extracted from cultured HUVECs using a Genomic DNA Purification Kit (K0512, Thermo Scientific™) according to the manufacturer's instructions. The concentration of DNA was quantified using a nucleic acid concentration detector (NanoDrop 2000, Thermo Scientific™). DNA samples were loaded on a polyvinylidencefluoride (PVDF) membrane (ISEQ00010, Millipore) using a 96-well dot-blot apparatus (1706545, Bio-Rad). The DNA-containing membrane was heated at 80 °C for 30 min and blocked with 5% nonfat milk for 1 h at room temperature. Then, the membrane was incubated in a monoclonal anti-5hmC antibody (A-1018-100, Epigentek) or anti-5mC antibody (A-1014-100, Epigentek) at 4 °C overnight. The corresponding secondary antibody (A0208, Beyotime) conjugated with peroxidase was applied to visualize 5hmC and 5mC. The densities of the dots were assessed using ImageJ image analysis software.

### Pyrosequencing assays

The pyrosequencing procedure has been described previously.^29^ Genomic DNA was extracted from cultured HUVECs and modified by bisulfite using the QiagenEpiTect Bisulfite Kit (59104, QIAGEN). The modified DNA was PCR-amplified using a PyroMark PCR Kit (978703, QIAGEN). Each amplicon was sequenced on a pyrosequencer (PyroMark Q96 ID, QIAGEN). The percentage of cytosine methylation within CpG dinucleotides was determined using the Pyro Q-AQ software. Amplification and sequencing primers were designed with PyroMark Assay Design 2.0.

### Statistical analysis

Statistical analyses were performed with Statistical Package for Social Sciences version 23.0. Data were presented as mean ± S.D. Statistical significance between two groups was evaluated with Student's *t* test (unpaired, two-tailed) and statistical significance among multiple groups were analyzed using ANOVA, followed by Bonferroni's multiple comparison test. The elongation of ECs was defined by the ratio of major axis to minor axis measured by ImageJ. All biochemical experiments and representative images were performed in at least three independent experiments.

## SUPPLEMENTARY MATERIAL

See the supplementary material for supplementary material figures (Figs. S1–S9).

## Data Availability

The data that support the findings of this study are available within the article and its supplementary material.
